# Tailoring of an anti-diabetic drug empagliflozin onto zinc oxide nanoparticles: characterization and in vitro evaluation of anti-hyperglycemic potential

**DOI:** 10.1038/s41598-024-52523-4

**Published:** 2024-01-30

**Authors:** Abdullah Shoaib, Sammia Shahid, Sana Mansoor, Mohsin Javed, Shahid Iqbal, Sajid Mahmood, Ali Bahadur, Fadi Jaber, Matar Alshalwi

**Affiliations:** 1 Department of Chemistry, School of Science, University of Management and Technology, Lahore, 54770 Pakistan; 2 Nottingham Ningbo China Beacons of Excellence Research and Innovation Institute, University of Nottingham Ningbo China, Ningbo, 315100 China; 3 Functional Materials Group, Gulf University for Science and Technology, 32093 Mishref, Kuwait; 4 Department of Chemistry, College of Science, Mathematics, and Technology, Wenzhou-Kean University, Wenzhou, 325060 China; 5 Dorothy and George Hennings College of Science, Mathematics and Technology, Kean University, 1000 Morris Ave, Union, New Jersey, 07083 USA; 6 Department of Biomedical Engineering, Ajman University, Ajman, UAE; 7 Center of Medical and Bio-Allied Health Sciences Research, Ajman University, Ajman, UAE; 8 Department of Chemistry, College of Science, King Saud University, PO Box 2455, 11541 Riyadh, Saudi Arabia

**Keywords:** Biochemistry, Bioinorganic chemistry

## Abstract

Diabetes is a serious health issue that can be a great risk factor related to numerous physical problems. A class of drugs “Gliflozin” especially Sodium Glucose Co. Transporter 2 was inhibited by a novel drug, which is known as “empagliflozin”. While ZnO nanoparticles (NPs) had considerable promise for combating diabetes, it was employed in the treatment and management of type-2 diabetes mellitus. The new drug empagliflozin was initially incorporated into Zinc Oxide NPs in this study using the surface physio-sorption technique, and the degree of drug adsorption was assessed using the HPLC method. The tailored product was characterized by using the FTIR, EDX, Ultraviolet–Visible, XRD and SEM techniques. With an average particle size of 17 nm, SEM revealed mono-dispersion of NPs and sphere-like form. The Freundlich isotherm model best fits and explains the data for the physio-sorption investigation, which examined adsorption capabilities using adsorption isotherms. The enzymes α-amylase and α-glucosidase, which are involved in the human metabolism of carbohydrates, were used in the in-vitro anti-diabetic assays. It was discovered that the composite showed the highest levels of 81.72 and 92.77% inhibition of -α-amylase and -glucosidase at an absolute concentration of 1000 μg per ml with IC_50_ values of 30.6 μg per ml and 72 μg per ml.

## Introduction

A rise in blood sugar levels is a defining characteristic of the metabolic condition diabetes mellitus (hyperglycemia). Deficiency of insulin synthesis, inadequate action of insulin, or the combination of these two, makes it difficult for glucose to move along the blood to tissues. Blood sugar levels increase as a result, and glucose is also discharged in urine. Patients having type 2 diabetes face the consequences of both short and long-term sickness which causes the death too soon. Due to type 2 DM’s dominance, and sneaky early stages of diagnosis, particularly in low economic and developing nations such as Africa, individuals with this condition tend to have increased sickness and death rates^1^.

The range of nanoscopic sizes, which fall between individual molecules and their bulk partners as nanomaterials, is 1–100 nm. By seeing all these aspects, it was observed that these are of incredibly small size, they have distinctive qualities with a great surface area and high energy^2^. Metal oxide NPs exhibited significant physical as well as chemical properties^3,4^. Zinc oxide NPs are well known in a great number of fields for their extraordinary properties and applications. The rubber industry was the first to use ZnO NPs because they can make rubber composites waterproof^5^, increase the intensity and toughness of polymers, act as an anti-aging agent, and perform other functions. The distinctive nanomaterials behaving like semiconductors and piezoelectric materials are zinc oxide NPs^6^. ZnO NPs were considered a great matter of interest in the biomedical field as anti-cancer, anti-oxidative, anti-diabetic, as well as anti-inflammation agents. These NPs also helped in the delivery of drugs and bio-imaging applications^7–10^. Doping involves adding an ion that wasn’t initially present in the starting material into a crystal lattice. Modulating the energy band gap can be very helpful because it directly affects the photocatalytic capabilities of ZnO and the associated antibacterial activity. The fabrication methods for preparing stable ZnO NPs have advanced significantly over the years. The primary ones are solution-free mechano-chemical processes, chemical precipitation, sol–gel, solid-state pyrolysis, and biosynthesis^11,12^.

Metal-oxide nanoparticles gained the interest of researcher because of their distinct physico-chemical characteristics and high surface area to volume ratio, nanosized inorganic metal oxides exhibit amazing biological activities at remarkably low concentrations. They are thermally stable at low and high temperatures^13–16^. Additionally, a small number of them are thought to be nontoxic to normal, healthy cells and tissues; as a result, their special theranostics and curative value are very beneficial for the treatment of disease. Similarly, these metal oxides can be biosynthesized into a variety of nanostructures, including nanotubes, nanoflowers, nanorods, and nanobelts^17,18^. Zinc Oxide nanoparticles gained more attraction among researchers among various metal oxide NPs. Zinc is a vital trace element that is involved in the creation of proteins and nucleic acids as well as other enzymatic processes. As a result, ZnO NPs use appears to be safe for healthy cells while being potent enough to trigger apoptosis in malignant cells^3,19–22^. Zinc Oxide nanoparticles (ZnO NPs) sowed antidiabetic potential as it increased serum insulin levels and lowered blood glucose levels considerably. The gene expressions of insulin receptor A, GLUT-2, and glucokinase were also examined in diabetic livers because zinc transporters, which are important regulators of glucose metabolism, are also found in adipose tissues and the liver. In the liver, ZnO NPs dramatically increased glucokinase expression and activity.

ZnO NPs have the following effects highlighting their anti-diabetic potential (a) they boost insulin secretion and fortify the antioxidant defence system in pancreatic β-cells; (b) they lower blood glucose levels and enhance glucose tolerance; (c) they enhance insulin signaling and sensitivity as well as the uptake of glucose by the liver, skeletal muscle, and adipose tissue; (d) they prevent lipolysis in adipocytes and gluconeogenesis in hepatic cells. Conversely, when taken orally, the minuscule size of zinc oxide nanoparticles maximizes their surface area and receptor sites, improving the medicine’s bioavailability. The combined action of the drug and the NPs has a synergistic effect on the treatment of diabetes. The FDA has classified ZnO NPs as a type of safe material^23^. Zinc Oxide nanoparticles were combined with the drugs and the synergistic effect of NPs and Drugs was observed. Embaby et al. studied the ZnO-NPs or MIL treatments, and the results showed improvement with minor renal lesions and fibrosis. The parameters under study were improved more by the combination therapies than by anyone alone^24^. Zinc oxide nanoparticles, or ZnO NPs, are a novel zinc delivery technique that has been created and evaluated in streptozotocin-induced diabetic mice in recent years. Abd El-Megeed et al. reported the use of ZnO NPs in streptozotocin-induced diabetic mice^25^. There are still some drawbacks as there is a dearth of research on the following topics: (1) comparative evaluation of the biological benefits of zinc oxide nanoparticles versus other metal nanoparticles; (2) toxicity of zinc oxide nanoparticles towards biological systems is still a contentious issue (3) evidence-based randomized studies specifically examining therapeutic roles in enhancing anticancer, antibacterial, anti-inflammatory, and antidiabetic activities; and (4) lack of understanding of related animal studies regarding these properties.

Gliflozins, also known as inhibitors of sodium-glucose co-transporter-2 (SGLT2), are one of several types of anti-diabetic medications (ADAs) having a proven track record of curing type-2 diabetes (T_2_D)^1,26^. However, due to its unique properties, the mechanism of action which is not dependent on the insulin (promote glycos-uria), which, is most probably used to lower the glucose present in plasma, also improves various kinds of metabolisms and hemodynamic abnormalities that are CVD risk causing agents, gliflozins possess distinct properties from other ADAs^27,28^. For the administration of type-2 diabetes (T_2_D) in adults, empagliflozin. It is a successful and generally well-tolerated anti-hyperglycemic medication. Due to its low inherent risk of hypoglycemia and independent mechanism, Empagliflozin is used for the treatment by using it alone and in combination therapy with supplementary antidiabetic drugs. It shows the balancing mechanisms to recover glycemia control in T_2_D patients^29^.

The research work focuses on the nano-materials applications such as the tailoring of drugs and how the nanomaterials are increasing the Drugs’ ability to prevent diabetes when used to treat the disease. Besides this, there is the preparation method of zinc oxide nanomaterials and the tailoring of active drugs with nanomaterials for the enhancement of the efficacy of the active drug. The fabricated material was characterized by Ultraviolet–Visible, FTIR, EDX, XRD, and SEM techniques. The aim of the work was an in-vitro study to check the efficacy of the tailored product for anti-diabetic activity.

## Materials and methods

### Chemicals and reagents

The present study employed just analytical-grade compounds that weren’t further refined. ZnSO_4_⋅7H_2_O the precursor for ZnO NPs and NaHCO_3_ used for the preparation of the ZnO NPs was from Merck. Empagliflozin was the main drug was received from Pharmagen Limited, Pakistan, and Methanol used to provide a medium for the adsorption of the drug was from Honeywell, and Deionized water was used for washing purposes.

### Methods

#### Synthesis of ZnO NPs

One gram of zinc-sulfate heptahydrate ZnSO_4_⋅7H_2_O was dissolved in 30 ml of bi-distilled de-ionized water, before being magnetically stirred to complete the process. Sodium carbonate solution, NaHCO_3_ (2 M) was added drop by drop until pH 7 approaches followed by continuous stirring.

**Figure 1:**
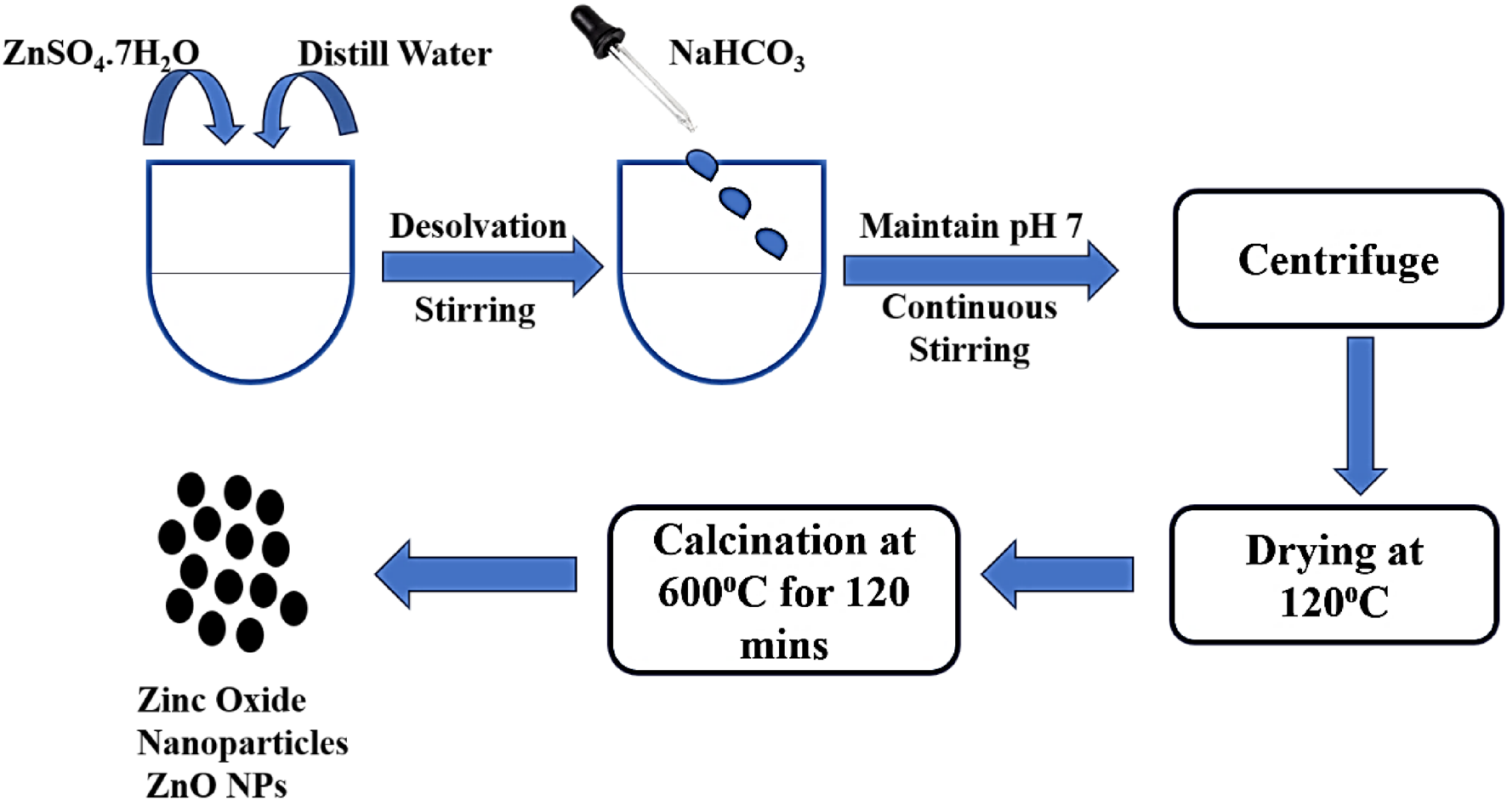
Synthesis scheme of ZnO NPs.

The final product underwent additional agitation for 1 h, followed by centrifugation and washing. After that, the sample was dried at 120 °C for about 12 h. The result was then ground up such that it could pass through meshes before being put into a white crucible for calcination at 600 °C for 120 min at a particular rate of 10 °C per minute^30^. The ZnO NPs powder was obtained and used for further characterization as shown below in Fig. 1. Table 1 represents the different variables for preparation.

**Table 1:** Different variables for preparation.

1. Concentration	1 g in 30 ml
2. Concentration of base	2 M
3. pH	7
4. Temperature	30 °C
5. Stirring speed	600 RPM
6. Addition time	1 ml in 5 min
7. Centrifugation speed	2000 RPM
8. Centrifugation time	10 min
9. Drying temperature	120 °C
10. Drying time	12 h

#### Adsorption of empagliflozin by ZnO NPs

**Figure 2:**
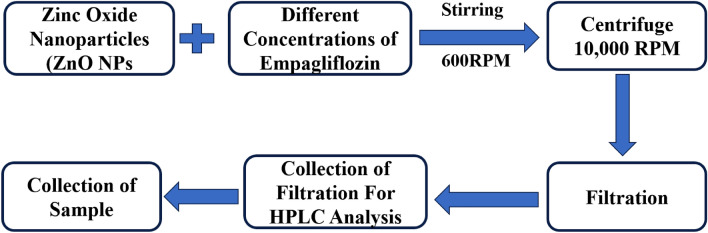
Schematic illustration for the adsorption of empagliflozin by ZnO NPs.

The drug was absorbed by the physio-sorption batch adsorption method. Empagliflozin was dissolved in absolute alcohol to make an exact starting concentration of 1.00 mg per ml which is considered as the stock solution. The concentration of the drug which indicates the maximum elimination percentage was then determined by doing serial dilutions of empagliflozin with various concentrations (0.1, 0.2, 0.3, 0.4, and 0.5 mg per ml) by using the stock solution. The whole procedure was conducted by using the solution taken in an Erlenmeyer conical flask of 100 ml at room temperature (25 °C). Then the final solution was stirred for 5 h at a fixed stirring rate of 600 RPM using 100 mg of ZnO NPs. The solution was then filtered using a vacuum pump and 0.45 m nylon filter paper after being centrifuged at 10,000 rpm. The previously described HPLC technique was used to assess the concentration of unbound empagliflozin as illustrated in Fig. 2^31^.

To evaluate the resilience of the technique repeatability following the standards for quality control and method validation, the adsorption performance for each experiment was repeated three times (as standard without ZnO NPs and evaluated with ZnO NPs. Three experimental replicates were used to get the data, which were then averaged. The drug removal percentage denoted by P (%), is the amount that was loaded and regarded as the “adsorption capacity” at equilibrium, denoted by the qe (mg/g), and was calculated using the following formulas.

$${\text{Percentage of drug}}\;{\text{in mg/l conc}}{.} = \frac{{\text{Areaof drug after loading}}}{{\text{Conc of drugs without loading}}} \times \frac{{\text{Area of drug without loading}}}{{\text{Conc of drug after loading}}} \times {\text{Percentage of drugs after loading}},$$

$${\text{Drug Removed }} = {1}00 \, - {\text{ Percentage of Drug adsorbed }}\% ,$$

$${\text{Amount of drug}}\;{\text{adsorbed }}\left( {{\text{qe}}} \right){\text{ mg/g}} = \left( {{\text{Initial concentrations of drug }} - {\text{ Equilibrium concentration of drug}}} \right) \, \times {\text{The volume of the drug/Mass of ZnO NPs }} = {\text{ Amount of Drug}}.$$

#### Characterization of ZnO NPs

For the comprehensive identification of the compounds, ultraviolet (UV) spectroscopy is carried out using a UV instrument by Shimadzu UV-1800 Japan. UV and visible spectroscopy followed the principle of “Beer’s–Lambert Law”. To identify phases, quantify powders and thin films while providing details on crystallinity, crystalline size, and orientation. Using an X-ray diffraction spectrometer (Maker Malvern Panalytical) worked at 40 kV at normal room temperature. X-ray diffraction spectra at the angle of 2θ ranging from 10° to 80° were obtained at normal temperature and 40 mA with Cu Kα radiation (1.54 Å)^32^. Morphology, topography, and chemical composition related to phase elucidation were studied with scanning electron microscopy at length scales ranging from nanometers to millimeters. The Nova Nano SEM-450 “Field-Emission-Scanning-Electron-Microscope (FE-SEM)” was used to carry out SEM with EDX analysis. At a very low voltage (1 kV) with a great minimum resolution of 1.4 nm within the great vacuum environment through the “TLD” Detector which was only operated within a very low vacuum. The FTIR spectroscopy technique is used for the analysis of the structure of compounds. FTIR analysis is performed for the spectroscopic study of the substances by focusing on the different kinds of bonds and as well as the functional groups within the substance by placing a tablet of the KBR having the material of interest on the front of the IR radiation^33^. By using an FTIR instrument IR-Prestige 21 makes Shimadzu and a spectrum within the range of 400 cm^−1^ to 4000 cm^−1^ is recorded covering the functional and fingerprint regions in which the presence of functional group with the modes of vibrations are studied and used for the basic identification of the whole molecule^34^.

#### In-vitro antidiabetic activity

##### Inhibition of α-amylase

For the preparation of the stock solution, a 10 ml volumetric flask was taken and 10 mg of the sample was added followed by the addition of 2 ml of alcohol to dissolve in it then make up the volume to mark with alcohol. The 1 mg/ml solution is equal to 1000 µg/ml which is considered as stock solution and then serial dilution of 750, 500, 250, 100, and 50.0 µg per ml of the solutions were prepared from the already prepared stock solution. To prepare the buffer solution having pH 6.9, 0.36 g of disodium hydrogen phosphate, 0.24 g of sodium dihydrogen phosphate, and 0.04 g of sodium chloride were poured into the volumetric flask and filled with 100 ml of distilled water and dissolved it. To prepare the 2 unit α-amylase solution 0.004 g of α-amylase was dissolved into 100 ml of buffer solution and after complete dissolution kept in the refrigerator. To prepare the 0.5% of starch solution 0.05 g of water-soluble starch was taken in 10 ml of hot boiling water and gently shaken to dissolve. To prepare the 3, 5-Dinitrosalicyclic acid (DNS) solution 0.1 g of DNS acid was taken and 0.5 N sodium hydroxide was added to it and heated to dissolve. 3 g of sodium potassium tartrate was added and dissolved by sonication and made up the volume to 10 ml with 0.5 N NaOH solution. The potential of empagliflozin loaded on ZnO NPs to inhibit the α-amylase enzyme was examined^35–37^.

##### Experimental design

**Figure 3:**
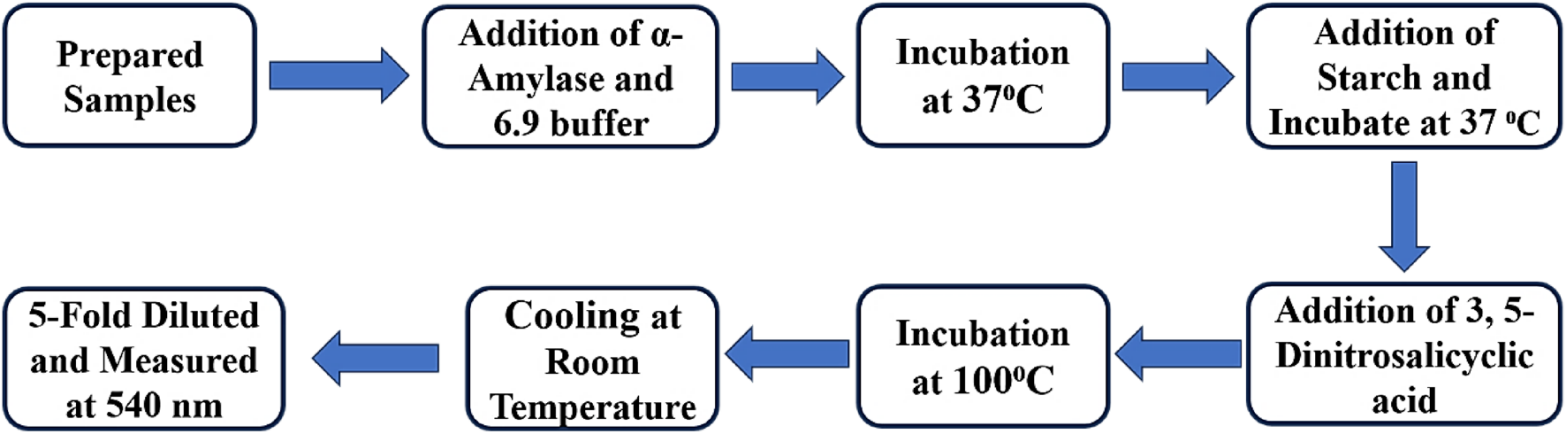
Inhibition activity of α-amylase.

50 μl of the above-mentioned sample at various concentrations (50.0, 100, 250, 500, 750, and 1000 μg/ml) were dropped to a 50 μl of an α-amylase solution containing (2 Units), and after adding the 1 mM phosphate buffer having (pH 6.9) the volume was increased to 250 μl. The sample solution was placed in an incubator at 37 °C for about 15 min. A starch solution with a concentration of (0.5%) was then added to the above mixture in a volume of 50 μl. It was again placed in an incubator for 15 min at 37 °C. Then a 500 μl DNS solution with the 0.1 M concentration was added to it. The synthesized product was then placed in a furnace at 100 °C in an oil-containing bath for 5 min before being cooled to room temperature. Then, at 540 nm, a sample mixture that had been diluted five times was found. In this study, Dapagliflozin served as a positive control. The catalyst was not used in the preparation of the control. Then proceed as mentioned in the sample solution as above in Fig. 3^38^.

$$\%age\, of\, \propto{\text{-}}amylase\, inhibition= \frac{Control-Sample}{Control} \times 100.$$

##### Inhibition of α-glucosidase

To prepare the 2-unit α-Glucosidase solution 0.004 g of α-glucosidase was poured into 100 ml of cool distilled water and dissolved and kept in the refrigerator. To prepare the 5 mM solution of nitrophenyl-β-d-glucopyranoside 0.015 g of nitrophenyl-β-d-glucopyranoside was added in cool distilled water and dissolved and kept on the refrigerator. To prepare the 0.1 M sodium carbonate solution 0.10 g of sodium-carbonate was taken in 10 ml of double distilled water and dissolved it. To prepare the Phosphate buffered saline solution, a described amount of Sodium chloride (NaCl), Na_2_HPO_4_, (NaH_2_PO_4_, KH_2_PO_4_ and KCl were taken and added to distilled water and dissolved^39,40^.

##### Experimental design

**Figure 4:**
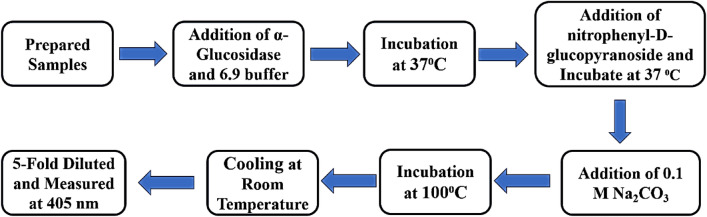
Inhibition activity of α-glucosidase.

The ZnO NPs’ ability to inhibit α-Glucosidase was assessed. For each sample, 25 µl of α-Glucosidase having (1 unit) was added to 50 µl of each sample at serial concentrations of 50, 100, 250, 500, 750, and 1000 g per ml followed by the addition of 1.0 mM phosphate buffer having (pH 6.9) and the volume was increased to 200 µl. After 15 min at 37 °C, the mixture was added to 50 µl of nitrophenyl-d-glucopyranoside (5 mM). The reaction mixture with the addition of substrate was placed in an incubator at the optimum temperature of 37 °C for about 15 min (Fig. 4). After the completion of the reaction, a 100 µl solution of Na_2_CO_3_ with a concentration of 0.1 M was dropped down into it to discontinue the reaction. The control used phosphate buffer saline having pH 6.9. Dapagliflozin was considered to work as a positive control. The resultant mixture was then analyzed using a UV–Vis spectrophotometer at 405 nm.

$$\%age\, of \,\propto{\text{-}}Glucosidase \, inhibition= \frac{Control-Sample}{Control} \times 100.$$

##### IC_50_ calculation

**Figure 5:**
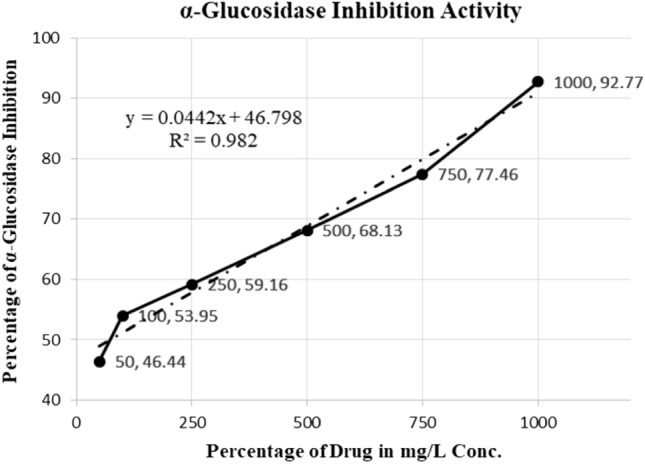
The IC_50_ value for glucosidase.

**Figure 6:**
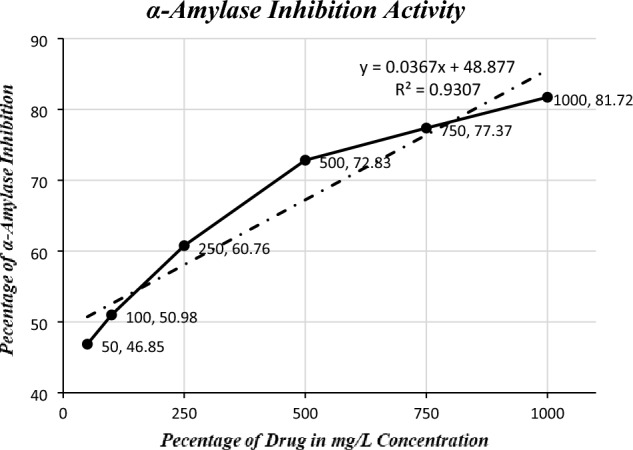
The IC_50_ value of α-amylase.

IC_50_ is the value of concentration at which 50% of the inhibitory activity has been completed. It is illustrated as the percentage of inhibition is drawn graphically with a concentration on the x-axis (Figs. 5, 6). When the Trend line was selected as an addition, a slope equation that could be used to determine the IC_50_ value appeared. After finding IC_50_ values for both the Standard and Sample, the concentration that prevented protein denaturation was noted (Fig. 6). The Formula used for the calculation of IC_50_ is

$${\text{Y}} = {\text{mx}} + {\text{C}}.$$

The IC_50_ value for Glucosidase can be calculated by using the above formula

$${5}0 \, = \, 0.{\text{442x }} + { 46}.{798},$$

$${5}0 \, {-}{ 46}.{798}/0.0{442 } = {\text{ x}},$$

$${\text{For Glucosidase}},{\text{ x }} = { 72}.{44 }\,\upmu {\text{g}}/{\text{mL,}}$$

$${5}0 \, = \, 0.0{\text{367x }} + { 48}.{877},$$

$${5}0 \, {-}{ 48}.{877}/0.0{367 } = {\text{ x}},$$

$${\text{For }}\alpha {\text{ - amylase x }} = { 3}0.{6 }\,\upmu {\text{g}}/{\text{mL}}.$$

## Results and discussion

### UV–Visible analysis

**Figure 7:**
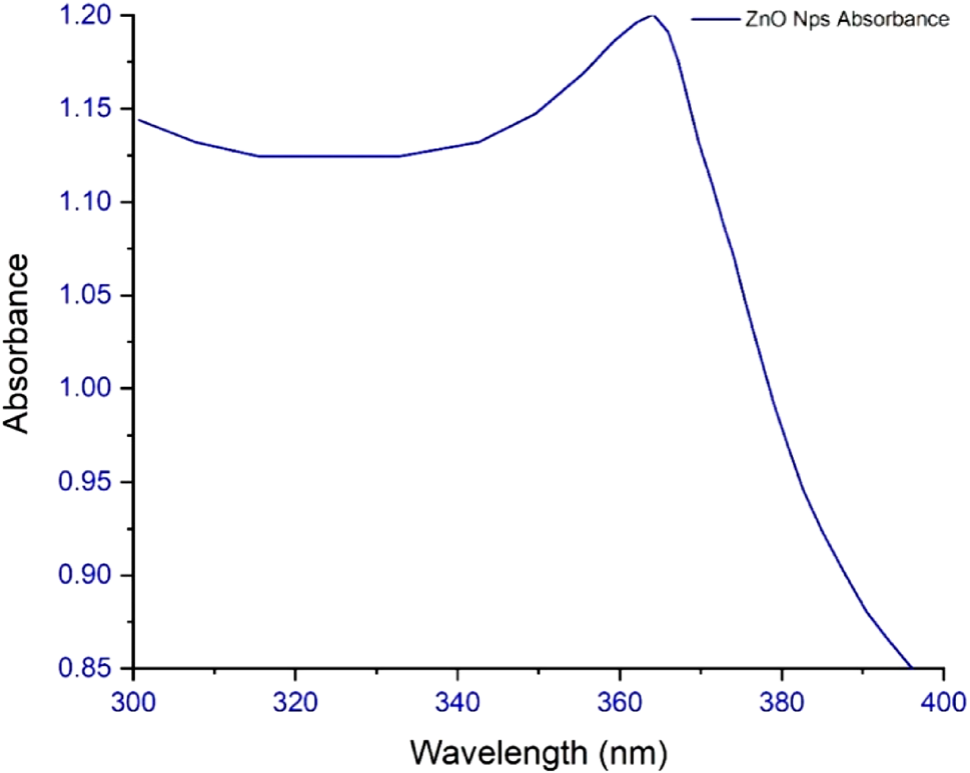
UV Analysis of ZnO NPs.

UV–Vis absorption spectra (Fig. 7) showed a maximum peak at 365 nm, which has confirmed that the material’s inherent band-gap absorption is generated when a transition is made by the outermost valance band electron to the conduction band (O_2_p → Zn_3d_). The band gap was calculated by using the equation,

$${\text{Eg }} = { 124}0/\lambda {\text{g}},$$

where ‘λg’ is the wavelength of absorption and results to be 3.40 eV for ZnO NPs^41^.

### FTIR analysis

**Figure 8:**
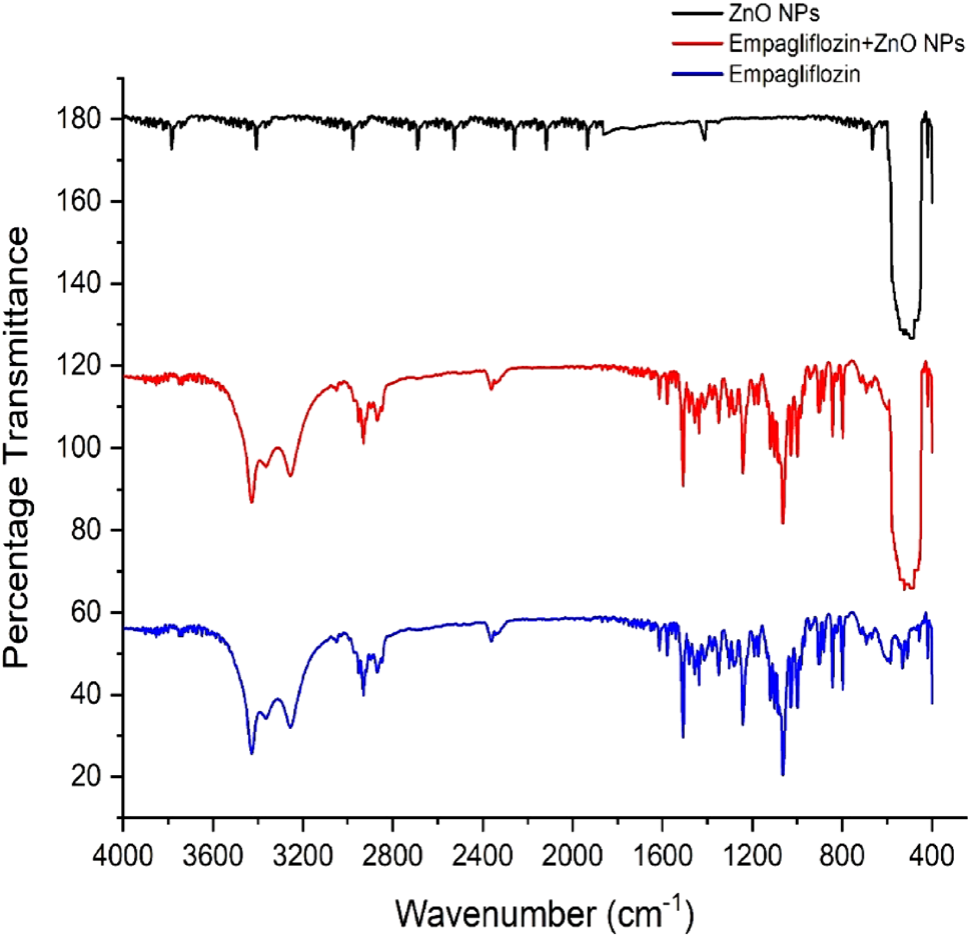
FTIR analysis of empagliflozin loaded ZnO NPs.

Using the FT-IR Spectroscopy technique, the exact chemical environment, bonding between the molecules, and cleanliness of the composite under study were verified. Figure 8 displays the given spectrum of the FT-IR transmittance, the resultant spectrums were of ZnO NPs, empagliflozin, and empagliflozin-ZnO-NPs in the 400–4000 cm^−1^ region at retention time. It was observed that the tested ZnO and Empagliflozin-ZnO NPs exhibited the wurtzite crystal structure of Zn–O NP’s transmittance at 430 and 445 cm^−1^ confirming the stretching modes of it. Some stretching modes are shown below the 500 cm^−1^ implying that ZnO-NPs were successfully produced^42^. The band with a core at 877–880 cm^−1^ is frequently discovered when the samples for FT-IR are studied without vacuum confirming the presence of an O–C–O bond which was the bending transmittance of carbonates^43^. The considerable transmittance bands at 3415–3415 cm^−1^ and 1625–1664 cm^−1^ were connected to the (O–H) stretching mode and (O–H–O) bending vibration by the water molecules that were absorbed on the surface, respectively^44^. The spectra obtained directly from the solid standard might be used to identify various empagliflozin distinguishing bands, as shown in Fig. 8. The FTIR spectra here is the research simply presenting the attachment of the drug with the nanoparticles by the physisorption mode not disturbing the actual chemistry of the concerned molecules.

The primary bands detected were those connected to the stretching of axial bonds and methyl group’s C–H bending, which were located in 2966–2985 cm^−1^ and 1367–1374 cm^−1^ ranges, correspondingly. Another prominent band in the spectrum includes those observed in the 1130–1225 cm^−1^ range, which appeared owing to absorption associated with stretching of C–O axial bonds. A very broad band between 1710 and 1722 cm^−1^ is due to the C=O group in the lactone^45^.

### XRD analysis

**Figure 9:**
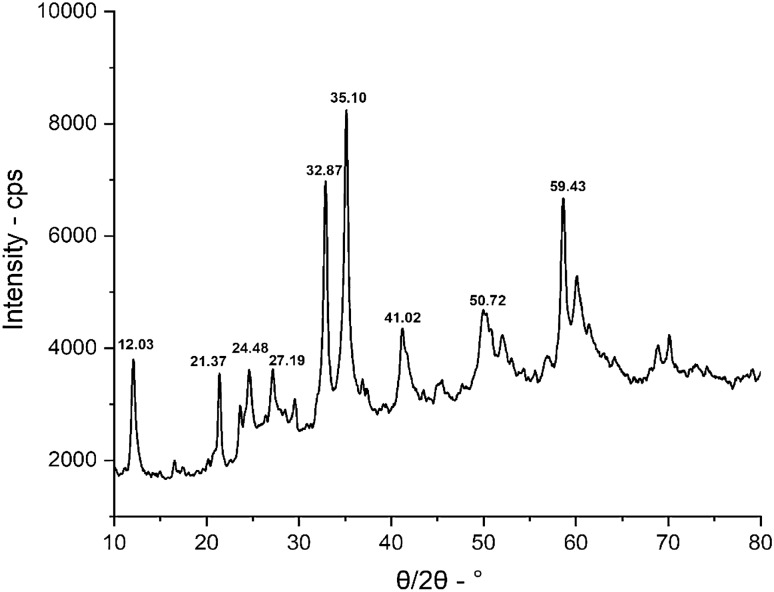
XRD analysis of empagliflozin loaded ZnO NPs.

The composite’s crystalline phase makeup of the fabricated Zn–O composite sample was evaluated using XRD analysis. Empagliflozin loaded ZnO NPs XRD patterns are displayed in Fig. 9 and XRD data is presented in Table 2. Strong and precise diffraction peaks were visible in the sample, which confirmed the extent of the degree of crystallization of Zn–O NPs. When ZnO is crystallized, its hexagonal (wurtzite) phase, no other crystalline phases associated with impurities could be found, demonstrating the formation of wurtzite crystal structure purity of the synthesized ZnO NPs. Additionally, the empagliflozin Loaded ZnO NPs sample’s maximum peak intensity was at 2θ = 35.105, It was coupled to the wurtzite ZnO phase via 101 Miller indices.

**Table 2:** Peak position & FWHM of empagliflozin loaded ZnO NPs.

Peak position (2 Theta)	FWHM	Crystal size D (nm)
12.03222	0.39579	20.1831
12.24336	0.96532	8.2769
21.37706	0.40216	20.1028
24.4859	1.5036	5.4065
27.19142	1.63688	4.9932
32.87422	0.54705	15.1408
35.10558	0.67439	12.3552
Average size (nm)		12.3512

The results were calculated by using Debye–Scherer formula, so, the ZnO sample’s exact average crystallite size denoted by (Dv) was measured.

$$Dv = \frac{0.94 \lambda }{\left(FWHM\right)\,{\text{cos}} \,\theta }.$$

FWHM value which was equal to the full width at half maximum expressed in radians (120 planes), diffraction angle denoted by the “ϴ” and λ was the wavelength of 1.5418 Å of the X-ray source. The crystallite size was found to be 12.35 nm.

### SEM analysis

**Figure 10:**
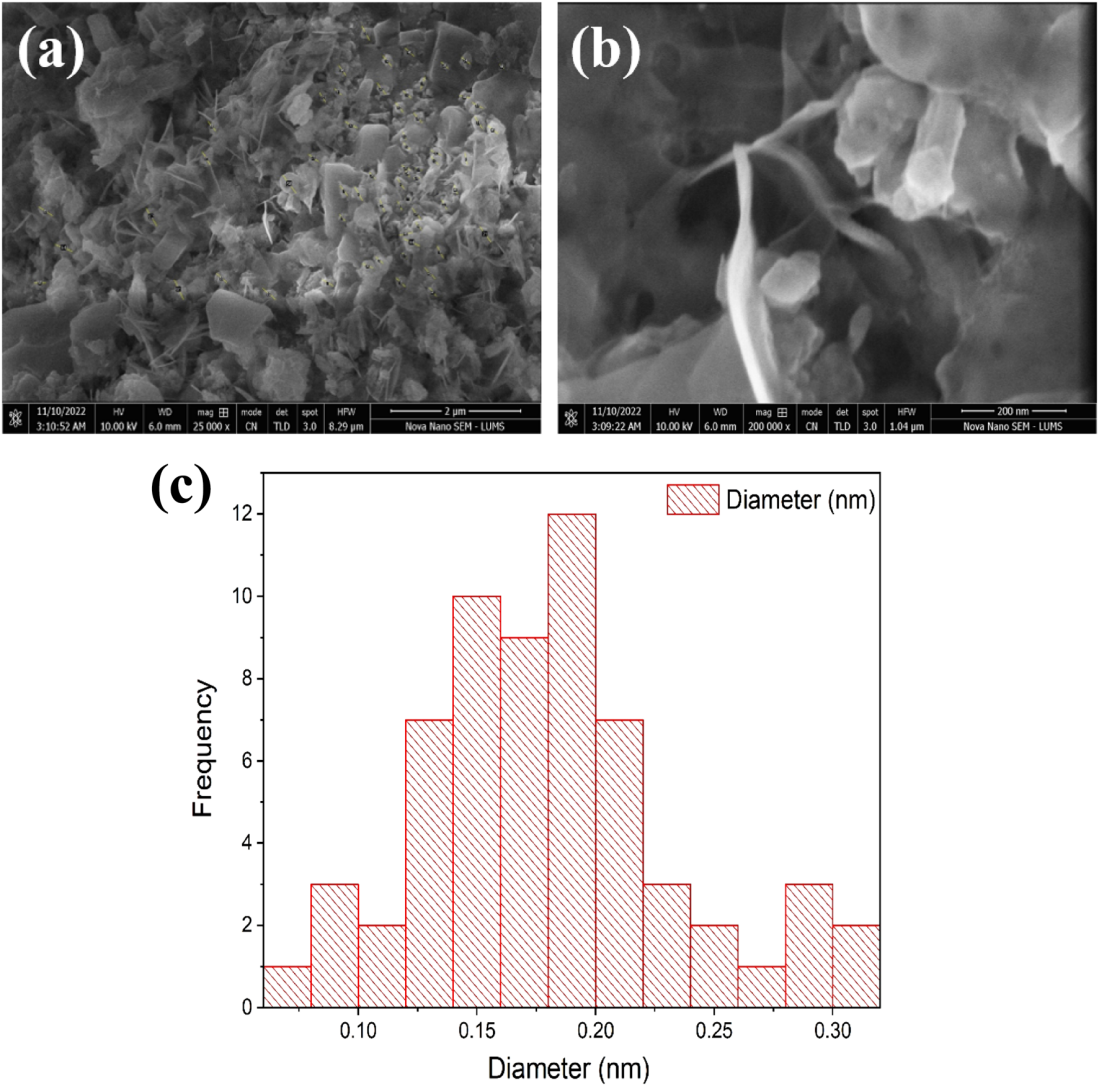
(**a**) SEM Analysis of Empagliflozin Loaded ZnO NPs and (**b**) SEM Analysis of Empagliflozin Loaded ZnO NPs at 200 nm. (**c**) Histogram of SEM Analysis of Empagliflozin Loaded ZnO NPs.

SEM investigation revealed that ZnO NPs were mono-dispersed and had a sphere-like form. The huge aggregation of nearly spherical forms with various sizes of a particle of the synthesized materials was visible in typical SEM micrographs of ZnO NPs and empagliflozin-ZnO samples, as illustrated in Fig. 10a. The adsorption of empagliflozin on ZnO NPs has no impact on the morphology of the particles. Additionally, Fig. 10b displays the matching diameter distribution histogram of the empagliflozin-ZnO and ZnO NPs samples, and it was discovered that the particle size was 17 nm. The SEM analysis confirmed the exact shape and size of the NPs as there was no change in the morphology of the particles. The size of the particles matters a lot as in the literature the NPs having a size less the 30 nm have better permeability as they can move into and out of the blood membranes but the NPs having greater size cannot move in or out of the membranes due to their large size (Fig. 10c). The NPs with an ideal size of 17 nm can possess the better permeability of the drug due to the large surface area with maximum adsorption and attachment sites^46^.

### EDX analysis

**Figure 11:**
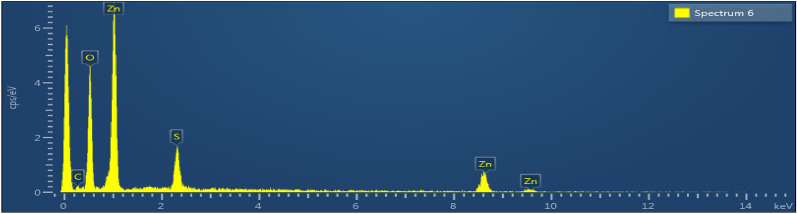
EDAX analysis of empagliflozin loaded ZnO NPs.

The chemical makeup of the Zn–O NPs sample was examined using EDX spectroscopy to assess the sample’s elemental and compositional stoichiometry properties (EDXS). Figure 11 depicts the EDAX spectrum of pure and mixed ZnO nanostructure samples. The produced powder samples show great purity, which was validated by the measured EDAX spectra, which showed that it was made entirely of zinc (Zn) and oxygen (O) in the absence of impurities.

### Adsorption of empagliflozin by ZnO NPs

The multilayer adsorption capacity of ZnO NPs was studied through the adsorption isotherms. It provides information about which kind of adsorption was taking place and according to the manifested data of isotherms a heterogeneous kind of adsorption process was observed. Moreover, the physisorption kind of attachment of Drug and ZnO NPs was observed. Table 3 shows how drug concentration affects the effectiveness of elimination. When the concentration of the drug is between 100 and 200 mg per l, the empagliflozin elimination dropped to 55.01 from 59.76%. The decline in the elimination was brought on by the concentration of the drug and the adsorbent’s vacated sites. Up until a saturation condition was reached, the drug adsorbed at high concentrations on the adsorbent’s vacant site. The clearance percentage significantly fell from 46.80 to 45.40% while the concentration of the drug increased from 300 to 500 mg/l. The decline can be ignored, though. After that the real concentration of the drug was 300 mg per Liter, percentage of drugs removed based on drug concentration also showed a consistent trend.

**Table 3:** Adsorption of Empagliflozin by ZnO NPs.

Percentage of drug in mg/l conc.	Percentage of drugs after loading (%)	Percentage of drug adsorbed (%)	Amount of drug adsorbed (qe) (mg/g)
Percentage of drug in 100 mg/l conc.	40.24	59.76	360
Percentage of drug in 200 mg/l conc.	44.99	55.01	320
Percentage of drug in 300 mg/l conc.	53.20	46.80	280
Percentage of drug in 400 mg/l conc.	54.02	45.98	240
Percentage of drug in 500 mg/l conc.	54.60	45.40	200

### Adsorption isotherm studies

**Figure 12:**
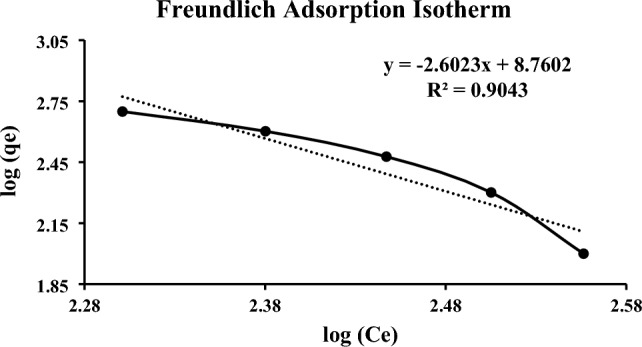
Freundlich adsorption isotherm.

The best model for design purposes was chosen by comparing two alternative isotherm models. According to Table 4, the isotherm calculations were completed. The Freundlich isotherm was found to be the isotherm model that best fits the data, as shown by the R^2^ values in Table 4 and Fig. 12. The adsorbent has a multilayer adsorption capability and is heterogeneous on the surface, according to the Freundlich model. According to the data revealed by the various employed isothermal models, certain crucial adsorption study parameters could also be studied, including the following. The following arrangement could be used to describe the adsorption isotherm of empagliflozin on the surface of ZnO NPs: Using the R^2^ value which is 0.904 as a parameter for indication, Freundlich > Langmuir.

**Table 4:** The research of the adsorption of Empagliflozin using ZnO NPs used several equations of the isothermal models.

No.	Equation	Parameter indication
01	Empagliflozin adsorbed amount qe = (C_0_ − Ce)V/M	qe: The adsorbed amount of EMPA (mg/g) C_0_: Initial concentrations of EMPA (mg/l) Ce: Equilibrium concentration of EMPA (mg/l) V: Volume of the EMPA (ml) M: Mass of ZnONPs (mg)
02	EMPA adsorption percentage P (%) = (C_0_ − Ce)/C_0_ × 100	
03	Langmuir Ce/qe = (1/q_L_K_L_) + (1/q_L_)Ce R_L_ = 1/(1 + K_L_Cmax)	q_L_: “monolayer adsorption capacity” ZnONPs (mg/g) K_L_: “Langmuir energy of adsorption constant” (l/mg) R_L_: sensitive equilibrium parameter or separation factor Cmax: “Highest initial drug concentration” in the solution (mg/l)
04	Freundlich log qe = log K_F_ + intensity (1/n) log Ce	K_F_: “Freundlich adsorption capacity” ZnONPs (mg/g) n: “Freundlich constant” characteristics of the system, indicating the intensity of adsorption

The n parameter, which was found to be (3.84) between 1 and 10, revealed that the Freundlich model identified an adsorption type that was preferred and necessary. Additionally, it was discovered that the value of 1/n, at 0.541, was less than the unit, indicating that the ZnO NP surface exhibits heterogeneity in the adsorption process. The experimental qe (200.0 mg/g) is discovered to be more than qL (160.4 mg/g), as shown by the Langmuir model. The RL, which was greater than 0 and less than the unity and was found to be 0.196, also supported the positive adsorption process. A value of 77.2 was observed and it is suggested that the value must be less than 80 and the resulting value was within the range. The fact that it was under 80 suggests it was best expressed in terms of physisorption kind of adsorption.

### In vitro anti-diabetic assays

The metabolism of carbohydrates is mediated by the two principal enzymes, α-amylase and α-Glucosidase. Increased blood sugar levels after meals in type 2 diabetes are significantly reduced by attenuating both enzyme activities. It has been shown that natural products significantly reduce the activity of digestive enzymes. Therefore, in this work, samples of ZnO NPs coated with empagliflozin were assessed for their capacity to inhibit enzymes. This study looked at the ZnO NPs laden with empagliflozin inhibitory effect against α-amylase. The highest α-α-amylase inhibitory activity was seen in the samples of Empagliflozin-loaded ZnO NPs at 1000 g/ml, whereas the lowest activity was seen at 50 g/ml (Table 5). Dapagliflozin, the positive control, significantly inhibited the activity of the enzyme amylase. The concentration of the samples of empagliflozin has a significant impact on the α-amylase inhibitory action. As a result, 5 µg/ml was discovered to be the IC_50_ concentration for α-amylase activity. In comparison to the other fractions^47^. Empagliflozin-loaded ZnO NPs showed the lowest IC_50_ concentration at 30.6 for the inhibition of α-amylase and significant interactions with Dapagliflozin. Additionally, Empagliflozin-loaded ZnO NPs’ ability to inhibit α-Glucosidase was discovered and is depicted in Table 6. The outcomes showed that ZnO NP with 1000 g/ml of empagliflozin loaded on it exhibits a considerable increase in α-Glucosidase inhibitory action compared to the other samples.

**Table 5:** α-Amylase inhibition activity.

Percentage of drug in mg/l conc.	Percentage of α-amylase inhibition (%)
Percentage α-amylase inhibition at 1000 µg/ml conc.	81.72
Percentage α-amylase inhibition at 750 µg/ml conc.	77.37
Percentage α-amylase inhibition at 500 µg/ml conc.	72.83
Percentage α-amylase inhibition at 250 µg/ml conc.	60.76
Percentage α-amylase inhibition at 100 µg/ml conc.	50.98
Percentage α-amylase inhibition at 50 µg/ml conc.	46.85

**Table 6:** α-Glucosidase inhibition activity.

Percentage of drug in mg/l conc.	Percentage of α-glucosidase inhibition (%)
Percentage α-glucosidase inhibition at 1000 µg/ml conc.	92.77
Percentage α-glucosidase inhibition at 750 µg/ml conc.	77.46
Percentage α-glucosidase inhibition at 500 µg/ml conc.	68.13
Percentage α-glucosidase inhibition at 250 µg/ml conc.	59.16
Percentage α-glucosidase inhibition at 100 µg/ml conc.	53.95
Percentage α-glucosidase inhibition at 50 µg/ml conc.	46.44

Significantly it was observed that the empagliflozin-loaded ZnO NPs reduce the activity that inhibits α-Glucosidase (Viladgliptin). Empagliflozin-loaded ZnO NPs were observed in 50 μg/ml samples and showed reduced enzyme inhibitory activity. Further, it was discovered that Dapagliflozin has an IC_50_ of 72.44 μg/ml for 1000 μg/ml against the α-Glucosidase. Among the remaining fractions, the empagliflozin-loaded ZnO NPs displayed the least IC_50_ concentration for α-Glucosidase and were significantly associated with Dapagliflozin (p < 0.05). According to the study, ZnO NPs loaded with empagliflozin inhibit α-Glucosidase as well as the α-amylase having IC_50_ concentrations of 5 μg per ml and 2.1 μg/ml, respectively^48–50^. Aside from that, earlier studies confirmed that dapagliflozin significantly inhibits the enzymes α-amylase and α-α-Glucosidase, having IC_50_ concentrations of 58.3 ± 0.8 µg per ml and 64.9 ± 1.1 µg per ml, respectively.

## Conclusion

The drug was then adsorbed using a batch adsorption approach with serial dilution after the ZnO NPs were made using the precipitation process. The study revealed that in a novel drug when tailored with ZnO NPs the efficiency of the drug was increased as the surface area and attachment level of the drug increased, and maximum adsorption was observed at the 100 mg/l solution concentration. The morphology, topology, particle size, and grain size distribution, as well as the elemental and identification of the ZnO NPs, were studied by SEM, XRD, FTIR, EDX and UV–Visible analysis, confirming the fabrication of ZnO NPs. A clear absorption maximum at 365 nm was observed in UV analysis and tailoring of zinc oxide was confirmed by the FTIR analysis as the clear transmittance at wavenumber 430 to 445 cm^−1^ is detected which showed the stretching type of vibrations. ZnO wurtzite crystal structure was depicted in the synthesized composite confirming the generation of ZnO NPs-empagliflozin composite with an average crystallite size of 17 nm. The SEM analysis and XRD analysis confirmed the wurtzite crystal structure formation with an average particle size distribution of 12.35 nm. Adsorption isotherms were studied and observed that the Frendulich isotherm was more suitable for the adsorption isotherms model. By the statistical analysis, it was observed that at the 100 mg/l concentration, 59.76% of the drug adsorption by the ZnO NPs displayed the maximum adsorption capacity of 360 mg/g of the drug. An in-vitro anti-diabetic assay with the two metabolic enzymes was performed at different concentrations and it was concluded that the tailored drug with the ZnO NPs showed great and enhanced anti-diabetic activity as the product induced 81.72% of α-amylase inhibition and 92.77% of α-Glucosidase inhibition showing the maximum and improved anti-diabetic activity. A dose of 1000 µg/ml of the tailored empagliflozin with ZnO NPs was sufficient to control diabetes in the patients suffering from hyperglycemia. Further studies will be required and in progress as analytical testing, stability studies, and the clinical efficiency of Tailored empagliflozin-ZnO NPs in the treatment of chronic diabetes.

## Data Availability

The datasets generated during and/or analyzed during the current study are available from the corresponding author upon reasonable request.
